# The Role of System-Specific Molecular Chaperones in the Maturation of Molybdoenzymes in Bacteria

**DOI:** 10.1155/2011/850924

**Published:** 2010-11-30

**Authors:** Meina Neumann, Silke Leimkühler

**Affiliations:** Department of Molecular Enzymology, Institute of Biochemistry and Biology, University of Potsdam, 14476 Potsdam, Germany

## Abstract

Biogenesis of prokaryotic molybdoenzymes is a complex process with the final step representing the insertion of a matured molybdenum cofactor (Moco) into a folded apoenzyme. Usually, specific chaperones of the XdhC family are required for the maturation of molybdoenzymes of the xanthine oxidase family in bacteria. Enzymes of the xanthine oxidase family are characterized to contain an equatorial sulfur ligand at the molybdenum center of Moco. This sulfur ligand is inserted into Moco while bound to the XdhC-like protein and before its insertion into the target enzyme. In addition, enzymes of the xanthine oxidase family bind either the molybdopterin (Mo-MPT) form of Moco or the modified molybdopterin cytosine dinucleotide cofactor (MCD). In both cases, only the matured cofactor is inserted by a proofreading process of XdhC. The roles of these specific XdhC-like chaperones during the biogenesis of enzymes of the xanthine oxidase family in bacteria are described.

## 1. Introduction

**Figure 1:**
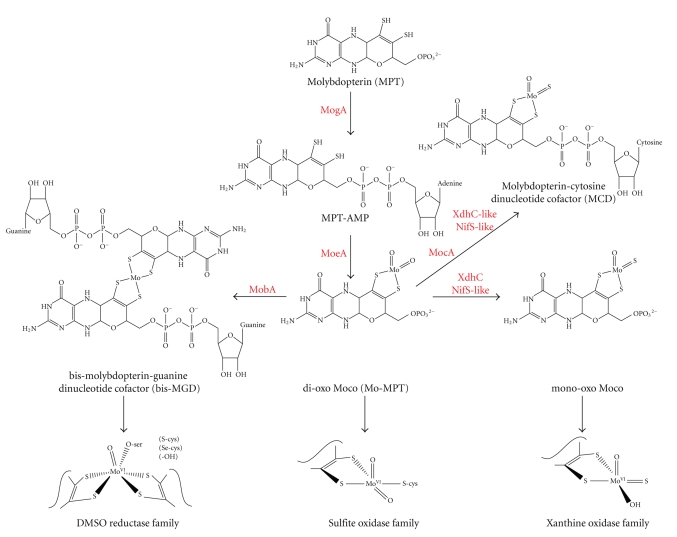
The biosynthesis of Moco and the families of molybdoenzymes. Shown is a scheme of the biosynthetic pathway of Moco from MPT in bacteria. The proteins involved in the reactions are colored red. In bacteria, Mo-MPT can be further modified by the MobA function which attaches GMP to the phosphate group of MPT, forming MGD, and two equivalents of MGD are bound to molybdenum, forming the so-called bis-MGD cofactor. For enzymes of the xanthine oxidase family, Mo-MPT or MCD (which is synthesized by the MocA protein by the attachment of CMP to Mo-MPT) can be modified by the replacement of one oxo-ligand by a sulfur ligand while forming the mono-oxo Moco. This reaction is catalyzed by a NifS-like protein in bacteria. The three molybdenum-containing enzyme families are divided into the DMSO reductase, the sulfite oxidase, and the xanthine oxidase families according to their active-site structures. The molybdenum center is shown in its oxidized state as Mo^VI^. Moco is the general term for all different variants of the cofactor.

Molybdenum is a transition metal that is incorporated as a biologically active cofactor (molybdenum cofactor, Moco) in a class of widely distributed proteins collectively known as molybdoenzymes [1]. Moco is associated with a wide range of redox enzymes and is found in most organisms from bacteria to humans. The metal in Moco is coordinated to a pterin derivative called molybdopterin to form the molybdenum-containing molybdopterin (Mo-MPT) cofactor [2]. A wide variety of transformations are catalyzed by these enzymes at carbon, sulfur, and nitrogen atoms, which include the transfer of an oxygen group or two electrons to or from the substrate. The mononuclear molybdenum enzymes are categorized on the basis of the structures of their molybdenum centers, dividing them into three families, each with a distinct active site structure and a distinct type of reaction catalyzed: the xanthine oxidase family, the sulfite oxidase family, and the DMSO reductase family [1] (Figure 1). The xanthine oxidase family is characterized by an LMo^VI^OS(OH) core in the oxidized state, with one equivalent of the pterin cofactor (designated L) coordinated to the metal. These enzymes (including xanthine dehydrogenase [XDH] and xanthine oxidase [XO]) typically catalyze the hydroxylation of carbon centers [1]. Enzymes of the sulfite oxidase family coordinate a single equivalent of the pterin cofactor with an LMo^VI^O_2_(S-Cys) core in its oxidized state (the cysteine ligand is provided by the polypeptide) [1]. Members of this family (including sulfite oxidase and plant nitrate reductase) catalyze the transfer of an oxygen atom either to or from the substrate. The DMSO reductase family is diverse in both structure and function, but all members have two equivalents of the pterin cofactor bound to the metal. The molybdenum coordination sphere is usually completed by a single M=O group with a sixth ligand in the L_2_M^VI^O(X) core [1]. The sixth ligand, X, can be a serine, a cysteine, a selenocysteine, or a hydroxide and/or water molecule. The reactions catalyzed by members of this family frequently involve oxygen-atom transfer, but dehydrogenation reactions also occur. While in bacteria, molybdoenzymes of all three families are present, eukaryotes only harbour molybdoenzymes of the xanthine oxidase or sulfite oxidase family. In bacteria, the basic form of Mo-MPT is generally modified by the attachment of CMP or GMP to Mo-MPT, forming the molybdopterin cytosine dinucleotide cofactor (MCD) or the bis-molybdopterin guanine dinucleotide cofactor (bis-MGD) (Figure 1) [3]. While the bis-MGD form of Moco is exclusively distributed in enzymes of the DMSO reductase family, the MCD cofactor was identified in bacterial enzymes of the xanthine oxidase family. In this family, enzymes contain either Mo-MPT or MCD with an equatorial sulfur ligand coordinated to the molybdenum center [4].

Enzymes of the xanthine oxidase family are the best characterized mononuclear molybdenum-containing enzymes. With a few exceptions, they catalyze the hydroxylation of different types of substrates according to the following reaction:

(1) $$\text{RH}+{\text{H}}_{2}\text{O}=\text{ROH}+2{\text{H}}^{+}+2{\text{e}}^{-}.$$

**Figure 2:**
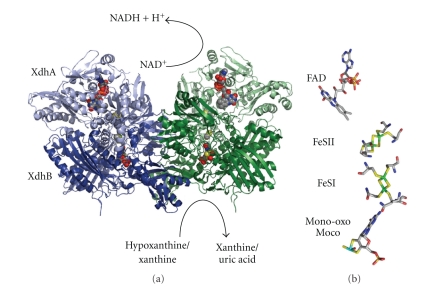
Overall structure and cofactor arrangement of *R. capsulatus* XDH. *R. capsulatus *XDH forms an (*αβ*)_2_ heterotetramer. The XdhA subunits are drawn in light green and light blue and the XdhB subunits in dark green and dark blue. The [2Fe-2S] and FAD cofactors of XdhA and the Moco of XdhB are shown as space-filling models. The Moco is deeply buried in the XdhB subunit being only accessible through a substrate-binding channel. Also shown is the coordination of Moco and FeSI, FeSII and FAD at the active site of *R. capsulatus *XDH and the reaction catalyzed by XDH. The structures were generated using the coordinates from the Protein Data Bank (accession number 1JRO).

This reaction occurs at the molybdenum center, which is, after interaction with substrate, reduced from Mo(VI) to Mo(IV). The two reducing equivalents generated in the course of the reaction are then transferred to an external electron acceptor by means of an electron transfer process mediated by other redox cofactors present in the structure of the protein. The crystal structure of the eight members of this family has been reported which are *Desulfovibrio gigas* aldehyde oxidoreductase (DgAOR) [5], *Desulfovibrio desulfuricans* aldehyde oxidoreductase (DdAOR) [6], *Bos taurus* xanthine oxidoreductase (bXOR) [7], *Rhodobacter capsulatus* xanthine dehydrogenase (RcXDH, Figure 2) [8], *Pseudomonas putida* Quinoline 2-oxidoreductase (PpQOR) [9], *Thauera aromatica* 4-hydroxylbenzoyl-CoA reductase (Ta 4-HBCR) [10] and the carbon monoxide dehydrogenase (CODH) from *Oligotropha carboxidovorans *[11] and *Hydrogenophaga pseudoflava *[12]. While bXOR and RcXDH contain Mo-MPT, all other crystallized bacterial enzymes were identified to bind MCD [13]. The majority of these enzymes (with the exception of 4-Hydroxybenzoyl-CoA reductase) are complex metalloflavoproteins that contain two nonidentical [2Fe2S] clusters, FAD, and the Moco as catalytically acting units (Figure 2) [14]. By contrast, CODH, which catalyzes a different reaction to that shown in (1), the conversion of CO to CO_2_ without cleavage of the CH bond, shows an active site that has never been observed in molybdoenzymes containing the pterin cofactor [15]. This comprises a dinuclear heterometal [CuSMoO_2_] cluster in which Mo and Cu ions are bridged by a sulfur ligand. CO oxidation is a source of energy for a wide diversity of prokaryotes and is an important process within the global carbon cycle. The enzyme CODH catalyzes the oxidation of CO and water to produce carbon dioxide, two electrons, and two protons [15]. The electrons are transferred to an electron transfer chain and used to generate a proton gradient across the membrane.

Xanthine oxidoreductase (XOR; which can be splitted into XDH, EC 1.17.1.4, and XO, EC 1.17.3.2) and aldehyde oxidase (AO; EC 1.2.3.1) are the best characterized members of the xanthine oxidase family [14, 16]. XOR has been the object of many reports and has long been recognized as the key enzyme in the catabolism of purines (Figure 2), oxidizing hypoxanthine to xanthine and xanthine to the terminal catabolite, uric acid, with the concomitant reduction of NAD^+^ (XDH) or O_2_ (XO) [17, 18]. XORs are of considerable medical interest, since this enzyme system in humans is implicated in gout and hyperuricemia, and its activity is responsible for postischaemic reperfusion injury. In bacteria, uric acid in most cases is further degraded to urea which is hydrolyzed to NH_4_^+^ and CO_2_ and thus can serve as a nitrogen and/or carbon source [19]. It was shown that *R. capsulatus* has the ability to utilize purines as sole nitrogen source [19]. While the biochemical function of XOR is well established, the biochemical and physiological function of AO is still largely obscure. The overall level of similarity between AO and XOR proteins is approximately 50%, which clearly indicates that the two proteins originated from a common ancestral precursor [14]. In humans, single monogenic deficits for any AO have not been described yet. AO is characterized by broad substrate specificity, and this makes it an important enzyme in the metabolism of drugs and xenobiotics. The physiological role of DgAOR was proposed to be linked to the degradation of polyglucose [5]. Also, in contrast to the other molybdo-flavoenzymes, DgAOR lacks the FAD domain [5]. Bacterial AOR can have a preference for aromatic aldehydes, as shown for the aldehyde oxidoreductase PaoABC from *E. coli* [20]. The physiological role of PaoABC in *E. coli* has been suggested to catalyze the detoxification of aromatic aldehydes to their corresponding less toxic acids in the periplasm [20]. 

The crystal structures of several molybdoenzymes revealed that Moco is deeply buried inside the proteins, at the end of a funnel-shaped passage giving access only to the substrate [21] (Figure 2). This implied the requirement of specific chaperones for each molybdoenzyme, to facilitate the insertion of Moco. For *R. capsulatus* XDH, the XdhC protein has not only been identified to be involved in Moco binding and formation of the terminal sulfur ligand, but was also shown to be involved in the final folding and maturation of apo-XDH after Moco insertion [22]. In prokaryotes, a number of specific chaperones were identified, also for members of the DMSO reductase family, like TorD for *E. coli* and trimethylamine oxide (TMAO) reductase (TorA) or NarJ for *E. coli* nitrate reductase A (NarGHI) [23–26]. This paper will focus on the molecular chaperones involved in the maturation of enzymes from the xanthine oxidase family in bacteria. At the end, a short comparison to the eukaryotic system is presented.

## 2. In Bacterial Genomes, Structural Genes Encoding for Members of the Xanthine Oxidase Family and Molecular Chaperones Are Clustered

**Figure 3:**
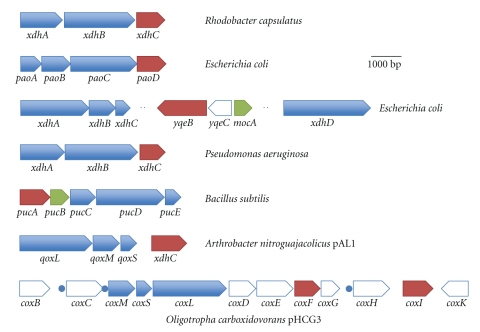
Schematic overview and organisation of operons including *xdhC*-like genes. Shown is the operon organization of different operons encoding for molybdoenzymes of the xanthine oxidase family of different bacteria. Operons of characterized molybdoenzymes are marked in blue, highlighted in red are the predicted genes encoding XdhC homologues, marked in green are genes for putative MocA-homologues, and marked in white are open reading frames encoding proteins with different (or not assigned) roles. In the operon structure encoding for the CODH, blue dots indicate predicted promoter sequences as published by Santiago et al. [27].

For *R. capsulatus* XDH, two genes, *xdhA* and *xdhB,* encode the polypeptides of the active enzyme [19]. XdhA was shown to bind the FAD cofactor and two [2Fe2S] clusters, and XdhB was shown to bind Mo-MPT (Figure 2) [8]. Immediately downstream of *xdhB*, a third gene was identified, designated *xdhC*, which is cotranscribed with *xdhAB* (Figure 3). Interposon mutagenesis revealed that the *xdhC *gene product is required for XDH activity [22]. However, XdhC is not a subunit of active XDH, which forms an (*αβ*)_2_ heterodimer in *R. capsulatus* (Figure 2). It was shown that XdhC neither is a transcriptional regulator for *xdh *gene expression nor influences XDH stability. The absence of Mo-MPT from XDH isolated from an *R. capsulatus xdhC *mutant strain indicated that XdhC might be a specific chaperone facilitating the insertion of Mo-MPT into XDH [22].

Genes similar to *R. capsulatus xdhC* have also been identified in a number of other prokaryotes. In some cases, a similar operon organization compared to the one present for *R. capsulatus* XDH was identified. However, so far, *R. capsulatus* XdhC is the only member of this family which has been characterized both on the genetic level and on the protein level [28].

*Pseudomonas aeruginosa *contains an* xdh* operon consisting of the genes *xdhABC*, like the one identified in *R. capsulatus* (Figure 3). Here, also *xdhAB* code for the structural genes of XDH with a similar subunit composition as identified for *R. capsulatus* XDH. An essential role of *P. aeruginosa* XdhC for XDH has not been demonstrated yet, for example, by interposon mutagenesis or on the purified proteins. However, for *P. aeruginosa* XdhC, it was shown that coexpression with the *xdhAB* structural genes from *Comamonas acidovorans* in a heterologous system in *E. coli* resulted in the production of an active XDH [29]. In the absence of *P. aeruginosa* XdhC, a lower Mo-MPT content was identified in the purified protein [29]. This suggested a similar role for *P. aeruginosa* XdhC in Mo-MPT insertion and XDH maturation as shown for *R. capsulatus* XdhC. 

In *E. coli*, the gene cluster for a periplasmic aldehyde oxidoreductase consists of *paoABCD* (Figure 3). The *paoABC* genes code for the three subunits of the trimeric aldehyde oxidoreductase, with PaoA binding two distinct [2Fe2S] clusters, PaoB binding FAD and PaoC containing MCD [20]. PaoD is predicted to be involved in MCD binding, maturation, and insertion into PaoC, since expression of *paoABC* in the absence of the *paoD* gene leads to inactive and instable PaoABC protein devoid of MCD [20]. Two additional open reading frames have been identified in *E. coli*, coding for XDH homologues, *xdhABC* and *xdhD* [19, 30, 31]. While both are predicted to have XDH activities, an XdhC homologue is not cotranscribed with these genes (Figure 3). However, a second XdhC homologue has been identified in *E. coli*, designated YqeB, which is organized in a single transcriptional unit in the genome. It remains to be elucidated whether YqeB is the XdhC-like chaperone shared by *E. coli* XdhD and XdhABC. 

**Figure 4:**
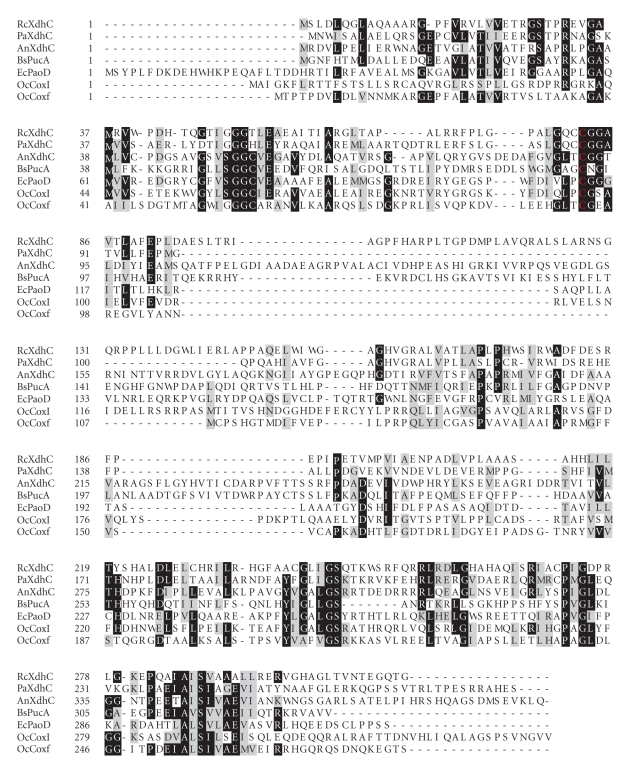
Amino acid sequence alignment of XdhC-like proteins from different bacteria. Shown is an amino acid sequence alignment of the XdhC-like proteins organized in operon structures (from Figure 1) from *R. capsulatus* (*Rc*XdhC), *E. coli* (*Ec*PaoD), *P. aeruginosa* (*Pa*XdhC), *O. carboxidovorans* (*Oc*CoxF, *Oc*CoxI), *A. nitroguajacolicus* (*An*XdhC), and *B. subtilis* (*Bs*PucA). A highly conserved cysteine residue present in all XdhC-like proteins is highlighted in red. Identical amino acids are boxed in black, and homologous amino acids are shaded in grey. The amino acid sequence alignment was performed using ClustalW and visualized using Boxshade.

Three genes, *coxL*, *coxM*, and *coxS *(for large, medium, and small subunits) encode the polypeptides for the CODH enzyme in *O. carboxidovorans *OM5 (Figure 3) [27]. Two heterotrimers, each composed of one CoxL, CoxM, and CoxS subunit, combine to form a functional aerobic CODH enzyme. The large subunit contains Moco, the medium subunit binds FAD, and the small subunit has two [2Fe2S] clusters [11]. In addition to these three genes, a number of other accessory genes have also been identified (CoxB, CoxC, CoxH, CoxD, CoxE, CoxF, CoxG, CoxI, and CoxK) that are believed to be required in the processes of regulation, posttranslational modification, and anchorage of the CODH complex to the cytoplasmic membrane. A number of these accessory genes are membrane-bound proteins themselves (CoxB, CoxC, CoxH, and CoxK), containing several transmembrane helices, and indeed, in *O. carboxidovorans *OM5, the CODH enzyme itself has been observed to be associated with the inner cytoplasmic membrane [27]. By sequence analysis, several promoters were annotated within the gene region containing 12 genes essential for carboxidotrophic utilization (Figure 3) [27]. CoxD was shown to be a MoxR-like AAA+ ATPase with a predicted function in the stepwise introduction of sulfur and copper in the [CuSMoO_2_] center of the enzyme [32]. The role of CoxF and CoxI was not characterized yet, but both proteins share amino acid homologies to *R. capsulatus* XdhC (Figure 4).

In contrast, the organization of the *B. subtilis puc* operon differs as the gene for the XdhC homologue, *pucA*, is located upstream of the structural genes *pucCDE* encoding for an XDH-like protein (Figure 3) [33]. Downstream of *pucA*, the *pucB* gene is located which encodes for a homologue to the CTP: molybdopterin cytidylyltransferase MocA involved in MCD biosynthesis (addition of the CMP moiety to Mo-MPT, [34]) (Figure 1). A knockout of *pucA *and *pucB* in* B. subtilis *decreased growth on hypoxanthine or guanosine as nitrogen source without affecting growth on ammonia or uric acid [33]. PucA has not been purified or characterized on the molecular level so far.

In *Arthrobacter nitroguajacolicus, *a linear catabolic plasmid containing a total of 103 open reading frames was shown to be responsible for quinaldine degradation [35]. The plasmid contains the operon *qoxMLS* encoding for the MCD-containing quinaldine dehydrogenase (QoxM-binding MCD, QoxL-binding FAD, and QoxS-binding 2 [2Fe2S] cluster). An XdhC homologue is located immediately downstream of QoxMLS [35]. The *qoxMLS* genes for quinaldine dehydrogenase were cloned into an expression vector and introduced into *P. putida* KT2440, resulting in the production of an active quinaldine dehydrogenase [35]. This showed that the *P. putida* XdhC homologue was functionally capable to insert the MCD cofactor into *A. nitroguajacolicus *QoxM. Neither the *P. putida* nor the *A. nitroguajacolicus* XdhC homologues were characterized on the molecular level so far. 

Figure 4 shows an amino acid sequence alignment of the XdhC-like proteins encoded by these operon structures. From the amino acid sequence alignment, it becomes clear that the overall sequence identity of the XdhC-like proteins from different bacteria is not high (~15%–30%). There are only very few highly conserved amino acids which are present in all XdhC-like proteins. However, one cysteine residue is conserved through almost all XdhC-like proteins present in the database, which corresponds to cysteine 82 in *R. capsulatus* XdhC (highlighted in red in Figure 4).

## 3. A Phylogenetic Point of View of the XdhC Family

**Figure 5:**
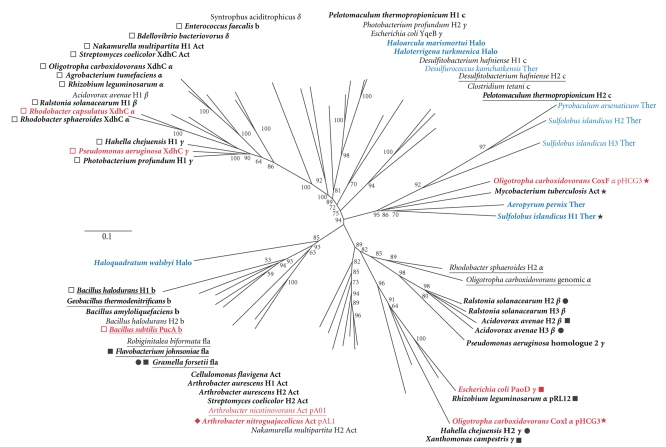
Phylogenetic tree of XdhC homologues. Protein phylogeny of XdhC homologues based on a full length sequence alignment. The tree was constructed by the neighbor-joining method from a matrix of estimated numbers of amino acid substitutions per site calculated with the Dayhoff option of Phylip. Numbers near branches indicate the bootstrap proportion for 100 replicas using the same method. The scale bar indicates 0.1 substitutions per site. Bacterial XdhC homologues from (partially) characterized operons are marked in red, XdhC homologues from Archaea are marked in blue, and in case several XdhC homologues present in one genome, they are marked by H1, H2, H3; class: b: Bacilli; c: Clostridia; *α*: Alphaproteobacteria; *β*: Betaproteobacteria; *γ*: Gammaproteobacteria; *δ*: Deltaproteobacteria; Act: Actinobacteria; fla: Flavobacteria; Halo: Halobacteria; Ther: Thermoprotei. Underlined XdhC homologues are part of an operon encoding Moco biosynthesis proteins, XdhC homologues in bold are part of an operon encoding a molybdoenzyme, putative molybdoenzyme partners were annotated based on a full length sequence alignment of the Moco binding subunit and by comparison of active site amino acids: PaoC homologues (filled square), XOR homologues (open square), CoxL/CutL homologues (filled star), isoquinoline 1-oxidoreductase homologues (filled circle) and quinaldine 4-oxidase (filled diamond).

The XdhC family of proteins contains hundreds of members that are bacterial and archaeal proteins, but no homologous sequences are present in eukaryotic genomes. While several organisms such as *R. capsulatus* contain only a single XdhC homologue, a large number of organisms like *Rhodobacter sphaeroides* or *E. coli* contain two or more homologues to XdhC. XdhC homologues are also present on strain-specific megaplasmids required for metabolic pathways like the one identified for quinaldine degradation in *A. nitroguajacolicus* Rü61a [35]. As discussed above, *xdhC* genes can be present in operons structures in conjunction with their respective structural genes encoding a member of the xanthine oxidase family (Figure 5, marked in bold). In some organisms, a gene organization was identified where genes encoding for XdhC homologues are cotranscribed with genes essential for Moco biosynthesis (Figure 5, underlined). 

As obvious from the phylogenetic tree, XdhC homologues being part of XOR operons in different Proteo- and Actinobacteria are clustered. These include XdhC of *R. capsulatus* XDH and *P. aeruginosa* XDH, which both contain the Mo-MPT form of Moco. In contrast, PucA, the chaperone of *B. subtilis* XDH [33], forms a cluster with distinct XdhC homologues in different *Bacillus* strains. Sequence alignments and phylogenetic analysis of the Moco-containing subunits indicate that these XDH homologues likely contain the MCD form of Moco. Thus, Mo-MPT and MCD-containing XDHs and their respective chaperones might have originated from a common ancestor but have evolved separately during evolution.

The operon organization of the Moco chaperone and the molybdoenzyme partner, for example, like in *R. capsulatus xdhABC* [19, 22] or *E. coli paoABCD* [20], might indicate that XdhC and PaoD are the system-specific molecular chaperones for *R. capsulatus* XDH and *E. coli* AOR, respectively. Other XdhC homologues, including *E. coli* YqeB, which are not organized in an operon structure with a specific molybdoenzyme, might represent examples for Moco-inserting chaperones responsible for several molybdoenzyme partners. Examples include the flavobacterium *Gramella forsetii* where an XdhC homologue is a part of an operon containing two putative molybdoenzymes and several putative Moco biosynthesis proteins. A similar organization is present on the megaplasmid pA01 of *Arthrobacter nicotinovorans* which includes genes for nicotine dehydrogenase and the ketone dehydrogenase [36]. This megaplasmid contains a single *xdhC* gene which is cotranscribed with a *mocA *gene [37]. Since detailed analyses of the role of these XdhC homologues for several molybdoenzymes have not been analyzed in detail yet, a shared role of these chaperones for several molybdoenzymes remains speculative. 

A more complex organization is present in *O. carboxidovorans*. The *cox* gene cluster of *O. carboxidovorans* megaplasmid pHCG3 encodes for two XdhC homologues, *coxF* and *coxI* (Figure 3) [27]. While the exact role of these homologues remains to be elucidated [38], phylogenetic analysis shows that CoxF is more similar to XdhC homologues found in other putative CODH operons and CoxI shows the highest similarity to *E. coli* PaoD. Moreover, the genome of *O. carboxidovorans* contains two additional genomic homologues of XdhC, one cotranscribed with the genes for a putative XDH and the other located in an operon with a MocA homologue.

## 4. The Absence of XdhC Affects the Maturation of the Molybdopartner

To date, a detailed characterization has only been performed for *R. capsulatus* XdhC [22, 28]. To analyze the function of XdhC for XDH in *R. capsulatus*, inactive XDH was purified from an *R. capsulatus xdhC *mutant strain [22, 28]. Analysis of the molybdenum cofactor content of this enzyme demonstrated that in the absence of XdhC, no Mo-MPT cofactor was present in the XdhAB heterotetramer. In contrast, absorption spectra of inactive XDH isolated from the *xdhC *mutant revealed the presence of iron-sulfur clusters and flavin adenine dinucleotide, demonstrating that XdhC is not required for the insertion of these cofactors. In the absence of Mo-MPT, XdhC remained associated with the inactive XDH heterodimer devoid of Mo-MPT [22, 28]. In addition, a heterologous system for the expression of *R. capsulatus* XDH in *E. coli *was established [39]. Here, the *R. capsulatus xdhABC* genes were coexpressed in *E. coli, *which resulted in a production of a 100% active XDH containing a full complement of the equatorial Mo=S ligand [40]. In the absence of XdhC, Mo-MPT was inserted into XDH in *E. coli*; however, the content of the equatorial sulfur ligand of molybdenum was drastically reduced [28]. This implied that XdhC is involved in the maturation of Mo-MPT by the addition of the equatorial sulfur ligand to the cofactor prior to the insertion into the XDH apoenzyme (in case of *R. capsulatus*).

The *E. coli* strain used in this study contained a deletion of the *mobAB *genes involved in bis-MGD formation [41], which resulted in an accumulation of Mo-MPT to unphysiological high concentrations. Thus, it is believed that Mo-MPT is unspecifically inserted into *R. capsulatus* XDH in *E. coli* during expression likely due to the accumulation of Mo-MPT. Whether one of the *E. coli* XdhC-like proteins is capable of inserting Mo-MPT into *R. capsulatus *XDH has not been investigated so far. In *E. coli*, the level of Moco sulfuration drastically depended on the oxygen concentration, since the amount of sulfurated Moco in XDH largely decreased with a high aeration of the expression cultures [28]. This implied a protective role of XdhC during Moco sulfuration. Similar results were also reported for *C. acidovorans *XDH expressed in *E. coli *[29]. 

For the characterization of XdhC, the protein was purified after heterologous expression in *E. coli *[28]. Characterization of the purified protein showed that XdhC is a dimer in solution and the purified protein is able to bind stoichiometric amounts of Mo-MPT or MPT, with dissociation constants of 3.6 ± 0.1  *μ*M and 3.5 ± 0.3  *μ*M, respectively [28]. It was also shown that XdhC-bound Mo-MPT can be inserted into Moco-free apo-XDH. Analysis of protein-protein interactions showed that XdhC specifically interacts with the XdhB subunit of XDH for Mo-MPT insertion [28]. The tight interaction of XdhC with XdhB underlined the specific role of XdhC in XDH maturation. For analysis of the role of the highly conserved cysteine residue Cys82 in *R. capsulatus* XdhC, site-directed mutagenesis was performed to exchange Cys82 by alanine. Unfortunately, all attempts to purify the XdhC-C82A variant failed, since this protein variant was highly unstable and precipitated completely during purification (unpublished results).

## 5. Formation of Sulfurated Moco

The investigations showed that XdhC binds Mo-MPT, which is produced by MoeA/MogA in the cell [42, 43] and protects it from oxidation until the terminal sulfur ligand is inserted. So far, proteins with a specific Moco sulfurase activity were identified in a number of eukaryotes. The first report was given by Wahl et al. [44] describing a mutation in the *Drosophila melanogaster maroon*-like locus (*ma-l*) which impaired the activity of XDH and AO, while the activity of sulfite oxidase remained unaffected [45]. Eukaryotic Moco sulfurase are two-domain proteins with an N-terminal domain showing homologies to bacterial L-cysteine desulfurase (E.C. 2.8.1.7) of the NifS family and a C-terminal domain with Moco-binding properties [46, 47], however, without homologies to the bacterial XdhC family. In general, L-cysteine desulfurase are homodimeric proteins that utilize pyridoxal 5-phosphate (PLP) to catalyze the reductive elimination of sulfur from L-cysteine, resulting in the formation of alanine and an enzyme-bound cysteine persulfide intermediate [48]. *R. capsulatus* and *E. coli* contain several L-cysteine desulfurase which could act as a sulfur donor for the sulfuration of Moco in these bacterial systems. The *E. coli* L-cysteine desulfurase are IscS, CsdA, and SufS [49], and the *R. capsulatus* homologues were named NifS (specific for nitrogenase), NifS2, NifS3, and NifS4 [50]. 

A specific role for the three *R. capsulatus* L-cysteine desulfurase NifS2, NifS3, and NifS4 had not been assigned before. Interposon mutagenesis of the *nifS2*, *nifS3*, and *nifS4 *genes showed that *nifS2 *strains displayed no particular XDH phenotype and that *nifS3 *and *nifS4 *are apparently essential for the viability of *R. capsulatus *[50]. To identify the specific L-cysteine desulfurase for the sulfuration of Moco in *R. capsulatus*, a direct *E. coli *two-hybrid screen was applied and the detected interaction partners were verified by surface plasmon resonance (SPR) measurements using the purified proteins. The L-cysteine desulfurase NifS4 of *R. capsulatus* was identified to specifically interact with XdhC with a dissociation constant of 0.64  *μ*M [50]. It was identified that NifS4 sulfurates Moco while it is bound to XdhC. An interaction of NifS4 with XDH was excluded. Thus, the XdhC-NifS4 pair can be considered as the prokaryotic counterpart to specific Moco sulfatases identified in eukaryotes. However, since interposon mutagenesis of *nifS4 *was not successful, the corresponding protein appears to have an additional role in another essential sulfur transfer pathway in the cell. NifS4 mobilizes sulfur from L-cysteine by formation of a protein-bound persulfide intermediate and transfers this sulfur further to XdhC-bound Moco. This reaction was shown to be more effective than the chemical sulfuration of Moco using sulfide as sulfur source. Further studies clearly showed that Moco is sulfurated before the insertion into XDH, while it is bound to XdhC.

## 6. A Model for the Insertion of Sulfurated Moco and Maturation of Molybdoenzymes

**Figure 6:**
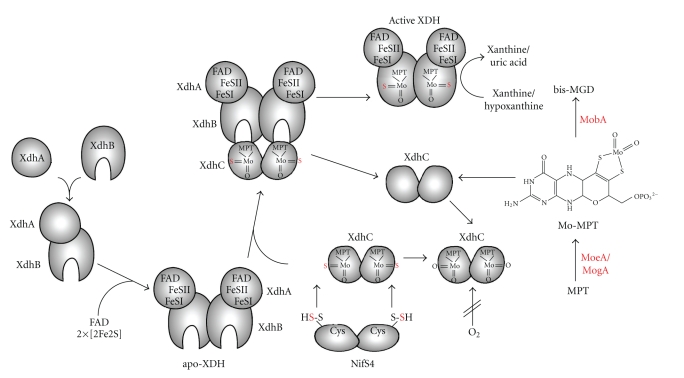
Model for the role of XdhC in the assembly of *R. capsulatus* XDH. The assembly of *R. capsulatus* XDH involves the synthesis of the XdhA and XdhB subunits, the dimerization of both subunits, the insertion of FeSI, FeSII, and FAD into the XdhA subunit, dimerization of two (*αβ*) dimers via the XdhB subunit to form the Moco-free apo-XDH, and finally, insertion of sulfurated Moco into XdhC. Mo-MPT is produced by molybdenum insertion into MPT catalyzed by MogA and MoeA and can further be converted to bis-MGD by the MobA protein. It is suggested that MoeA/MogA/MobA and XdhC form a complex in the cell. XdhC binds Mo-MPT, and the equatorial Mo=S is inserted into Moco before its incorporation into XDH by the sulfurtransferase function of the NifS4 protein. XdhC protects Moco from oxidation and interacts with XDH for Moco insertion.

The assembly of XDH is a highly ordered process, which involves the synthesis of the XdhA and XdhB subunits, the dimerization of both subunits, the insertion of FeSI, FeSII, and FAD into the XdhA subunit, the dimerization of two (*αβ*) dimers via the XdhB subunit, and finally, the insertion of sulfurated Moco into XdhB, resulting in an active enzyme (Figure 6) [51]. The biosynthesis of Moco is additionally a complex process involving more than a dozen different proteins [52, 53], with the insertion of sulfurated Moco by the XdhC protein being the last step of XDH maturation [22]. 

Moco is produced from MPT by the MogA-MoeA complex, catalyzing the ATP-dependent ligation of the molybdenum atom to MPT (Figure 6) [54, 55]. Synthesized Mo-MPT can subsequently be transferred either to MobA, converting it into bis-MGD (which is inserted into enzymes of the DMSO reductase family) [56], or to XdhC, forming the sulfurated form of Moco by exchange of an oxoligand with sulfur (which is inserted into XDH). XdhC interacts with MobA and thereby inhibits the transfer of Moco to MobA [43]. Further, it is believed that XdhC dissociates from this complex to interact with the L-cysteine desulfurase NifS4. NifS4 directly interacts with XdhC and exchanges the equatorial oxygen ligand specifically by a sulfur ligand. The sulfur for this reaction originated from L-cysteine and a persulfide group is formed on NifS4 in the course of the reaction. Further, XdhC has to dissociate from NifS4 after the sulfuration reaction to interact with XdhB. The present results for *R. capsulatus *XDH demonstrate that dimerization via the *β*-subunits is required to stabilize a structure of the protein that makes the protein suitable for Moco insertion [51]. Since XdhC is a dimer itself, it is suggested that the XdhC dimer interacts with the XdhB dimer simultaneously inserting matured and sulfurated Mo-MPT into both active sites of XdhB (Figure 6). The insertion of sulfurated Moco into XDH is strictly regulated in *R. capsulatus*, because *in vivo *dioxo-Moco is not inserted into XDH [28]. The mode of control of this step has not been identified to date. After this reaction, XDH is correctly folded and XdhC dissociates from the complex. Since XdhC is very labile in the absence of Moco, it is believed that after performing the insertion reaction, XdhC is degraded in the cell.

Thus, XdhC performs several control reactions: (i) to ensure that Moco is sulfurated by the interaction with the L-cysteine desulfurase NifS4 [50] before insertion into XDH and (ii) to insert the sulfurated Moco in the formed (*αβ*)_2_ heterotetramer of XDH. Because Moco is deeply buried in the protein, it is also believed that XdhC acts as a chaperone being involved in proper folding of XDH after Moco insertion [22]. The model shows that apo-XDH exists in a Moco competent “open” conformation until the insertion of sulfurated Moco and that after the insertion reaction, the protein adapts the final active “closed” conformation, which is incapable to accept Moco (Figure 6).

## 7. Functional XdhC Homologues in Eukaryotes

Eukaryotic Moco sulfurase are two-domain proteins containing one protein domain with homologies to L-cysteine desulfurase and a second protein domain belonging to a superfamily of *β*-strand-rich domains, named MOSC domains [57]. These MOSC domain proteins are characterized to contain an absolutely conserved cysteine residue. The MOSC domain was predicted to be a sulfur-carrier domain that receives the sulfur abstracted by the PLP-dependent L-cysteine desulfurase domain on its cysteine residue and delivers it for the formation of, for example, sulfurated Moco in case of the Moco sulfurase [57]. Bacterial homologues to the MOSC superfamily were also identified, like the *E. coli* YiiM protein [58], and one to four orthologs of YiiM per genome are present in diverse bacteria such as *α*- and *γ*-proteobacteria and gram-positive bacteria [57]. The *E. coli* YiiM protein was characterized to be involved in the detoxification of 6-N-hydroxylaminopurine to adenine, a pathway which depends on the presence of active Moco [58]. It could be clearly shown that the role of YiiM is distinct from the formation of sulfurated Moco for enzymes of the xanthine oxidase family [58]. However, the precise role and the involvement of Moco in the reaction remains to be elucidated for YiiM.

The currently best characterized eukaryotic Moco sulfurase is ABA3 from *Arabidopsis thaliana* [59, 60]. ABA3 is composed of an N-terminal NifS-like domain and a C-terminal MOSC domain. L-cysteine desulfurase activity and sulfur transferase activity of ABA3 were shown to be essential for the activity of AO, being involved in abscisic acid (ABA) biosynthesis in plants. It has been suggested that a persulfide sulfur is transferred from the NifS-like domain of ABA3 to the Moco in AO of plants [60]. More recent studies showed that the C-terminal MOSC domain is able to bind Moco in a 1 : 1 ratio with a dissociation constant of 0.55 ± 0.14  *μ*M, implying that the C-terminus of ABA3 represents the functional homologue of XdhC in bacteria [61]. In addition, it was shown that the Moco bound on ABA3 existed in its sulfurated form. However, in contrast to the case in bacteria, Wollers et al. [61] proposed that the role of the ABA3 C-terminus in activation of XDH and AO occurs posttranslationally, after the insertion of Mo-MPT in these enzymes. Evidence for this assumption was obtained from experiments showing that the activity of these enzymes in ABA3 C-terminus mutant extracts (*sir3-3*) was increased by a chemical, nonenzymatical sulfuration procedure. This implied that AO in plants is present in both a sulfo and a desulfo form, which can posttranslationally be activated by ABA3. Thus, in plants, AO and XDH remain inactive until one of the two oxygen ligands is replaced by a sulfur atom delivered by ABA3. By this mechanism, it was suggested that the plant is able to rapidly increase the activities of AO and XDH without *de novo* synthesis of the apoproteins, representing a mechanism of rapid posttranslational regulation [59, 61]. Unfortunately, the authors did not consider the possibility that in the *sir3-3 ABA3* mutant, the nonsulfurated Moco was inserted unspecifically into AO. Following the model by Bittner's group, it remains to be elucidated how AO and XDH receive their sulfurated Moco. In plant AO and XDH, this could occur either by an exchange of a sulfurated with a nonsulfurated Moco by aid of ABA3 or by exchange of solely the sulfide-ligand. Both models are largely different from the system in bacteria, where a quality control mechanism guarantees that only sulfurated Moco is inserted into enzymes of the xanthine oxidase family, and a posttranslational regulation of enzyme activity via its sulfuration level does not occur. In bacteria, after insertion of sulfurated Moco which is deeply buried into the enzyme, the enzyme is correctly folded. Thus, eukaryotic Moco sulfurase must have developed a system for the unfolding of the inactive holoenzyme to either extract the Moco from it or to sulfurate the Moco while bound to the target enzyme. This shows that for a similar function, the eukaryotic and the prokaryotic systems must have evolved independently.

## 8. Concluding Remarks

This paper highlights the key roles of the bacterial XdhC family of system-specific chaperones in Moco modification and insertion into enzymes of the xanthine oxidase family. Due to a lack of information on other XdhC-like proteins, this paper is mainly focussed on the role of *R. capsulatus* XdhC for XDH. However, on the basis of sequence similarities and gene organizations in other bacteria, we believe that *R. capsulatus* XdhC represents an example of a large family of XdhC-like proteins which perform the same function for their specific molybdoenzyme partners. These specific molecular XdhC-like chaperones are involved in the late steps of Moco biosynthesis by protecting Mo-MPT against oxidation for its final maturation step, the exchange of the equatorial oxygen ligand at the Mo center against a sulfide group. All XdhC-like proteins contain a highly conserved cysteine residue, which might perform a similar function as the conserved cysteine in the MOSC-like domains of eukaryotic Moco sulfatases. So far, the role of this cysteine residue is not completely clear in both systems. It could either act as a sulfur-transferring cysteine on which a persulfide is formed during the reaction, but it also could serve as a ligand to the protein-bound Mo-MPT, thus, either stabilizing the bound Moco or representing a ligand to generate an electron environment that facilitates the sulfur/oxygen exchange reaction. After synthesis of the mono-oxo Moco, the cofactor is specifically inserted into a target enzyme. Conclusively, the XdhC family of proteins must specifically recognize and interact with its target protein partner. During the maturation step of the apomolybdoenzyme, XdhC must stabilize a competent state of the apoenzyme for cofactor insertion. The matured Moco is inserted into the catalytic site of the enzyme which, in its final form, is deeply buried in the enzyme. After final folding of the molybdoenzyme, XdhC has performed its role and is no longer required for enzyme activity and consequently dissociates from the holomolybdoenzyme. In total, these XdhC proteins perform versatile roles for Moco and molybdoenzyme maturation, involving the interaction with different proteins, which has to be highly specific. Attempts to solve the crystal structure of XdhC are under investigation, which will shed light into the interaction sites and Moco binding site of this protein. In eukaryotes, functional homologues to XdhC exist, which have further evolved from this system and are fused to one interaction partner, the L-cysteine desulfurase which forms sulfurated Mo-MPT. Further investigations will clarify whether the C-terminal MOSC domain of eukaryotic Moco sulfurase performs a similar role like XdhC in bacteria. In total, the XdhC family represents an exquisite model to study the maturation of molybdoenzymes.
